# Influences of gestational diabetes mellitus on the oral microbiota in offspring from birth to 1 month old

**DOI:** 10.1186/s12884-022-04630-1

**Published:** 2022-04-06

**Authors:** Qiying Song, Bin Xiao, Hongli Huang, Liya Ma, Jian V. Zhang, Yuanfang Zhu

**Affiliations:** 1 Maternal-Fetal Medicine Institute, Shenzhen Baoan Women’s and Children’s Hospital, Jinan University, No.56 Yulv Road, Baoan, 518100 Shenzhen, China; 2 Center for Energy Metabolism and Reproduction, Institute of Biomedicine and Biotechnology, Shenzhen Institutes of Advanced Technology, Chinese Academy of Sciences, No.1068 Xueyuan Avenue, Nanshan, 518055 Shenzhen, China; 3 Shenzhen Luohu Maternity and Child Health Care Hospital, Luohu, Shenzhen, 518019 China; 4 Department of Child Healthcare, Shenzhen Baoan Women’s and Children’s Hospital, Jinan University, No.56 Yulv Road, Baoan, Shenzhen, 518100 China

**Keywords:** Gestational diabetes mellitus, Oral microbiota, Infancy, 16S rRNA

## Abstract

**Background:**

Maternal gestational diabetes mellitus (GDM) had long-term influences on the health of their children. However, the influences of GDM on the oral microbiota, which was closely related to oral and systemic health in offspring, were less documented. The present study aimed to explore the oral microbiota of neonates born to mothers with GDM is differentially colonized compared with those born to mothers without GDM, and whether any such differences persist to 1 month of age.

**Methods:**

Oral samples were collected from children of mothers with (*n* = 20) and without GDM (*n* = 34) at birth and again at an average age of 1 month. The oral microbiota was characterized by 16S rRNA sequencing (V3-V4). Differences in diversity and composition according to maternal GDM status were assessed, and different metabolic functional pathways and microbial ecological networks were also analyzed.

**Results:**

Although no significant differences were observed in diversity metrics between GDM and non-GDM groups (*P* > 0.05), we found significant differences in the taxonomic composition of oral microbiota from phylum to genus level between the two groups, with the GDM group exhibiting less abundance of *Veillonella* in both “Day 1” (*P* < 0.001) and “Day 30” (*P* < 0.05) phases. Metabolic pathways analysis showed that 5-aminoimidazole ribonucleotide biosynthesis and inosine-5'-phosphate biosynthesis were enriched in GDM subjects in the “Day 30” phase. Moreover, ecological network analysis revealed apparent differences between GDM and control groups, with the non-GDM group containing more high-degree nodes and microbial interactions compared with the GDM group.

**Conclusion:**

Maternal GDM was associated with an altered oral microbial composition in neonates, although the distinct difference between GDM and non-GDM groups diminished in infancy. The oral microbiota functions and ecological networks differed dramatically between the two groups, highlighting the importance of maternal GDM status on initial oral microbiota in offspring.

## Introduction

Gestational diabetes mellitus (GDM), defined as “any degree of glucose intolerance with onset or first recognition during pregnancy”, is a common medical complication of pregnancy [1]. The incidence of GDM is on the rise globally [2, 3], with an incidence of approximately 15% of all pregnancies in China [4]. Aside from the short-term maternal, fetal, and neonatal consequences associated with GDM, such as pre-eclampsia, preterm delivery, fetal macrosomia, and so on, there are long-lasting consequences for both mother and child, including increased risks of obesity, metabolic syndrome, and type 2 diabetes [2, 5]. Although compelling studies have been done to explore the potential links between GDM mothers and health consequences on their children, it still has not been fully understood.

In recent years, research has increasingly shown that GDM altered the gut microbiota composition in the offspring [6–9]. However, the influences of GDM on the oral microbiota of the offspring were less studied [10, 11]. He et al. [11] found a distinct oral microbiota profile in neonates born to mothers with GDM. Another study by Singh et al. [10] also revealed a significant difference in composition of the neonatal oral microbiota between GDM and control groups. However, both studies were possibly confounded by various perinatal conditions known to disrupt offspring oral microbiota colonization, such as maternal gestational weight gain, delivery mode, and gestational age. In addition, the oral sample collection was restrict to only one time point (i.e., 1 min after birth) in both studies with a cross-sectional design. Whether the differences in diversity and composition of oral microbiota according to maternal GDM status persist remains unclear.

Accumulating evidence demonstrates that a diverse array of early oral and systemic diseases were associated with the disruption of oral microbiota of children, including dental caries, celiac diseases, autism, pediatric appendicitis, and pediatric inflammatory bowel syndrome [12–14]. In addition, some studies revealed an interesting finding that the oral microbiome might relate to nitrate metabolism and cardiovascular health [12, 15, 16]. In order to better assess oral and systemic health and prevent future diseases at an early age, we should learn more about the establishment of the oral microbiome during early life.

Therefore, in the present study, with a well-controlled, hypothesis-directed study design, we aimed to: (a) investigate whether the oral microbiota of neonates of mothers with GDM is differentially colonized compared with neonates born to mothers without GDM; and (b) if any differences exist, explore whether the differences persist to 1 month of age, thus potentially contributing to the capability of using the oral microbiome to predict oral and systemic diseases later in life.

## Methods

### Participants

The present study was conducted at the Shenzhen Baoan Women's and Children's Hospital affiliated to Jinan University in 2021, and obtained approval from the Ethical Committees of Shenzhen Baoan Women's and Children's Hospital (approval number: LLSC 2020–09-02-KS). Written informed consent to participate in this study was provided by the children's parents or legal guardians. All methods were carried out in accordance with relevant guidelines and regulations.

A total of 54 full-term, normal birth weight newborns were included in the baseline and followed up to 1 month old, with 20 neonates born to mothers with GDM and 34 neonates born to mothers with normal gestational plasma glucose regulation. Newborns with any significant congenital anomaly, neurological dysfunction, fetal chromosomal abnormality, or metabolic diseases were excluded. None of the women had received antibiotic treatment during the whole pregnancy. Moreover, women with GDM were included only if they were managed by diet or exercise, without the use of medications (such as insulin or oral agents) to control blood glucose.

All women were offered a standardized 75-g oral glucose tolerance test (OGTT) between 24 and 28 weeks during pregnancy. Women were diagnosed with GDM if one or more of the following glucose criteria were met: fasting ≥ 5.1 mmol/L, 1 h ≥ 10.0 mmol/L, or 2 h ≥ 8.5 mmol/L, according to the International Association of the Diabetes and Pregnancy Study Group (IADPSG) criteria and WHO recommendations [1, 17].

Information on maternal and infant characteristics, including maternal age at delivery, pre-pregnancy BMI, gestational weight gain, delivery mode, infant’s sex, gestational age, birth weight, and length were derived from the electronic medical records of the hospital information system. In addition, information on feeding practices (breastfeeding, formula, or mixed), use of probiotics, weight and length at 1 month of age were also collected from the medical records. None of the infants received antibiotic treatment before the 1-month visit.

### Collection of oral samples

Two oral samples were collected by trained nurses for each included infant at birth (henceforward referred to as “D1 phase”) and again when they returned to the hospital for the 1-month visit (median = 30 days, range: 28 ~ 42 days, henceforward referred to as “D30 phase”). During the sample collection, nurses wore face masks and sterile gloves to avoid possible contaminations during the whole process. All oral samples were collected with sterile swabs and then placed in 1000μL of cell lysis solution immediately after collection and then stored in a -80℃ freezer for DNA extraction.

### Sequencing and sequence processing

Genomic DNA was extracted from oral samples using the MoBio PowerSoil DNA isolation kit (MoBio, Carlsbad, CA) according to the manufacturer’s instructions. Its concentration and quality were assessed using Qubit (Invitrogen) and verified with agarose gel electrophoresis. The 16 s rRNA gene was amplified using 338F 5’ACTCCTACGGGAGGCAGCAG3’ forward primer and 806R 5’GGACTACHVGGGTWTCTAAT3’ reverse primers targeting the V3-V4 hyper-variable regions. All quantified amplicons were equally pooled and sequenced on the Illumina MiSeq system (Illumina Inc., CA, USA) with the paired-end mode. Raw sequencing data were deposited into the NCBI Sequence Read Archive database (SRA BioProject ID: PRJNA760654).

16S rRNA sequences were analyzed using QIIME2 software (version 2020.11) [18]. After the sequencing reads were demultiplexed with a custom Perl script, the paired-end sequencing reads were imported into a QIIME2 artifact with the command “qiime tool import”. Then quality filtered, which involved the removal of Phix and the processing of chimeric sequences with the command “qiime dada2 denoise-paired”. The taxonomic assignment was finished with the command “qiime2 feature-classifier classify-sklearn” command against Greengenes (13_8 revision) database [19]. The indexes for alpha- and beta- diversity were generated with the command “qiime phylogeny align-to-tree-mafft-fasttree” and “qiime diversity core-metrics-phylogenetic” at a sample depth of 10,000 according to the tutorials of QIIME2 [18].

Finally, a total of 1,862,851 of 16S rRNA valid reads (mean reads per sample = 44,354; min to max = 22,384–74,134; SD = 10,174) were generated from oral samples in D1 phase, and 2,398,499 reads (mean reads per sample = 45,255; min to max = 35,887–60,565; SD = 5,967) from D30 phase.

### Statistical analysis

The statistical analysis was performed using R software (version 3.6.1). *P* value < 0.05 was considered statistically significant. Continuous characteristics were analyzed using unpaired *t*-tests and reported as means ± standard deviation (SD). Categorical data were studied with Fisher’s exact tests and presented as numbers and percentages to compare the differences in general characteristics between the GDM and non-GDM groups. For microbiota data, the alpha diversity metrics were compared by the Wilcoxon rank sum test. The weighted unifrac distance was applied to determine multivariate sample distances and visualized by principal coordinates analysis (PCoA). Permutation-based analysis of variance (PERMANOVA) was used to compute the difference of beta-diversity between GDM and non-GDM groups. At various taxonomic levels (from phylum to genus level) we performed generalized linear model (GLM) assuming a negative binomial distribution to determine the significant different microbes between offspring of mothers with and without GDM according to a previous study [9], addressing potential confounding by delivery mode, maternal pre-pregnancy BMI, gestational weight gain, infant sex, gestational age, and feeding practices. In addition, PICRUSt2.0 software (Phylogenetic Investigation of Communities by Reconstruction of Unobserved States) in QIIME2 was used to predict metabolic functional composition [20]. The different metabolic functional pathways between GDM and non-GDM groups were generated using the STAMP (v2.1.3) program with extended error bar plot [21], following with Benjamini–Hochberg correction. Furthermore, the microbial ecological network was analyzed using SPIEC-EASI software [22].

## Results

### Cohort characteristics

Fifty-four neonates (20 neonates born to mothers with GDM and 34 neonates born to mothers without GDM) were included and followed to 1 month of age. The maternal and infant characteristics between the GDM and non-GDM groups are presented in Table 1. Except that the glucose levels in fasting, 1 h post-OGTT, and 2 h post-OGTT were significantly higher in GDM women than in non-GDM women (*P* < 0.05), the other maternal characteristics, including age at delivery, pre-pregnancy BMI, gestational weight gain, and delivery mode did not differ between the two groups (*P* > 0.05). There were also no significant differences in infant characteristics at birth and at 1 month between the GDM and non-GDM groups (*P* > 0.05), indicating the general characteristics were similar in the two groups.

**Table 1:** Maternal and infant characteristics comparison between the GDM and non-GDM groups (*N* = 54)

	**GDM (*N* = 20)**	**Non-GDM (*N* = 34)**	***p* value**
**Maternal characteristics**			
Age at delivery, year	32.6 ± 4.3	30.5 ± 3.4	0.052
Pre-pregnancy BMI, kg/m2	23.3 ± 2.9	21.8 ± 3.5	0.109
Gestational weight gain, kg	12.5 ± 4.0	13.7 ± 4.8	0.366
Delivery mode			0.600
Vaginal	14 (70.0)	26 (76.5)	
Cesarean	6 (30.0)	8 (23.5)	
Fasting glucose, mg/dL	5.1 ± 0.9	4.3 ± 0.3	**0.005**
OGTT-1 h, mg/dL	9.9 ± 2.2	7.8 ± 1.3	**0.002**
OGTT-2 h, mg/dL	8.0 ± 2.2	6.4 ± 1.0	**0.011**
**Infant characteristics at birth**			
Sex, Male/female	8/12	19/15	0.260
Gestational age, week	39.2 ± 0.8	39.5 ± 1.0	0.167
Birth weight, kg	3.2 ± 0.5	3.2 ± 0.4	0.810
Birth length, cm	49.7 ± 1.8	50.1 ± 1.3	0.386
**Infant characteristics at 1 month**			
Age at collection of oral sample, days	31.4 ± 2.8	30.8 ± 1.4	0.419
Feeding practices			0.335
Breastfeeding	11 (55.0)	25 (73.5)	
Mixed	7 (35.0)	8 (23.5)	
Formula	1 (5.0)	1 (2.9)	
Missing	1 (5.0)	0 (0)	
Use of probiotics	1 (5.0)	1 (1.6)	1.000
Weight, kg	4.3 ± 0.6	4.4 ± 0.5	0.572
Length, cm	54.4 ± 2.1	55.0 ± 1.8	0.282

Data are shown as means (SD) or n (%). Significant differences in maternal and infant characteristics between the two groups are highlighted in bold

*Abbreviations*: *BMI* Body mass index, *GDM* Gestational diabetes mellitus, *OGTT* Oral glucose tolerance test

### Overall microbial community structures of oral microbiota

**Fig. 1:**
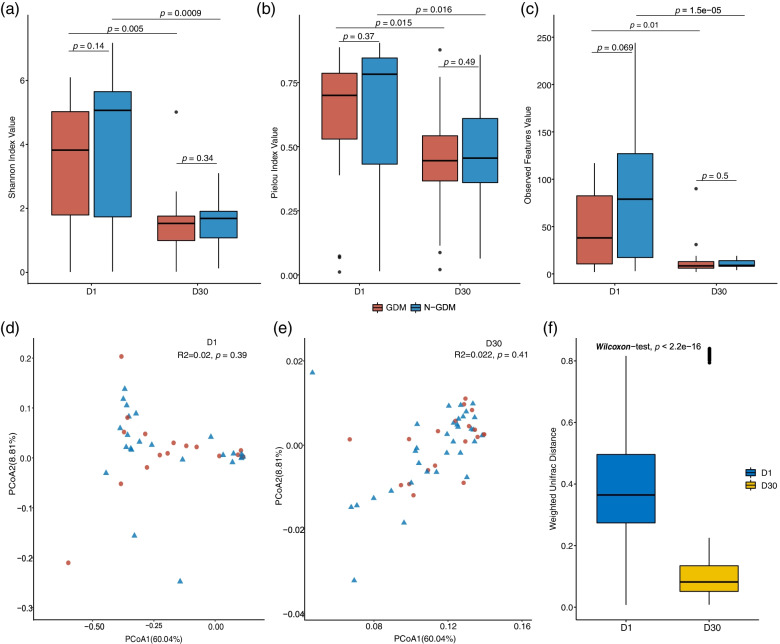
Alpha and beta diversity of oral microbiota in newborns of mothers with and without GDM. **a** Comparisons of Shannon’s index. **b** Comparisons of Pielou’s evenness. **c** Comparisons of observed features value. **d** Principal ordination analysis based on weighted UniFrac distances in the D1 phase. **e** Principal ordination analysis based on weighted UniFrac distances in the D30 phase. **f** comparison of weighed UniFrac distances between GDM and non-GDM groups in D1 and D30 phases

Compared with the non-GDM group, the average Shannon’s index values in the D1 phase (3.36 ± 2.01 vs 4.0 ± 2.33, *P* = 0.14) and D30 phase (1.53 ± 1.07 vs 1.62 ± 0.68, *P* = 0.34) were both slightly lower in the GDM group without significance (Fig. 1a). Similarly, slightly lower average values of Pielou’s index and observed features value were also observed in D1 (Pielou’s index: 0.60 ± 0.28 vs 0.63 ± 0.30, *P* = 0.37; observed features value: 49.72 ± 41.04 vs 84.83 ± 66.14, *P* = 0.069) and D30 (Pielou’s index: 0.44 ± 0.21 vs 0.49 ± 0.20, *P* = 0.49; observed features value: 13.95 ± 19.02 vs 10.76 ± 4.51, *P* = 0.50) in GDM group than in non-GDM group (Fig. 1b and c). Compared with the D1 phase, Shannon’s index, Pielou’s evenness, and observed features value all significantly decreased in the D30 phase, regardless of the GDM group or non-GDM group.

To compare the overall microbial structure in neonates born to mothers with or without GDM, PCoA was implemented based on the weighted UniFrac distance. The results showed no difference between GDM and non-GDM groups both in D1 and D30 phases (*P* = 0.39 and 0.41, respectively, Fig. 1d and e). However, the average value of weighted UniFrac distance between GDM and non-GDM group in the D30 phase was significantly lower (0.36 ± 0.15 vs 0.12 ± 0.17, *P* < 0.001, Fig. 1f) than that in the D1 phase, which indicated that the oral microbiome between the two groups became more and more similar over time.

### Taxonomic abundance in GDM and non-GDM groups

**Fig. 2:**
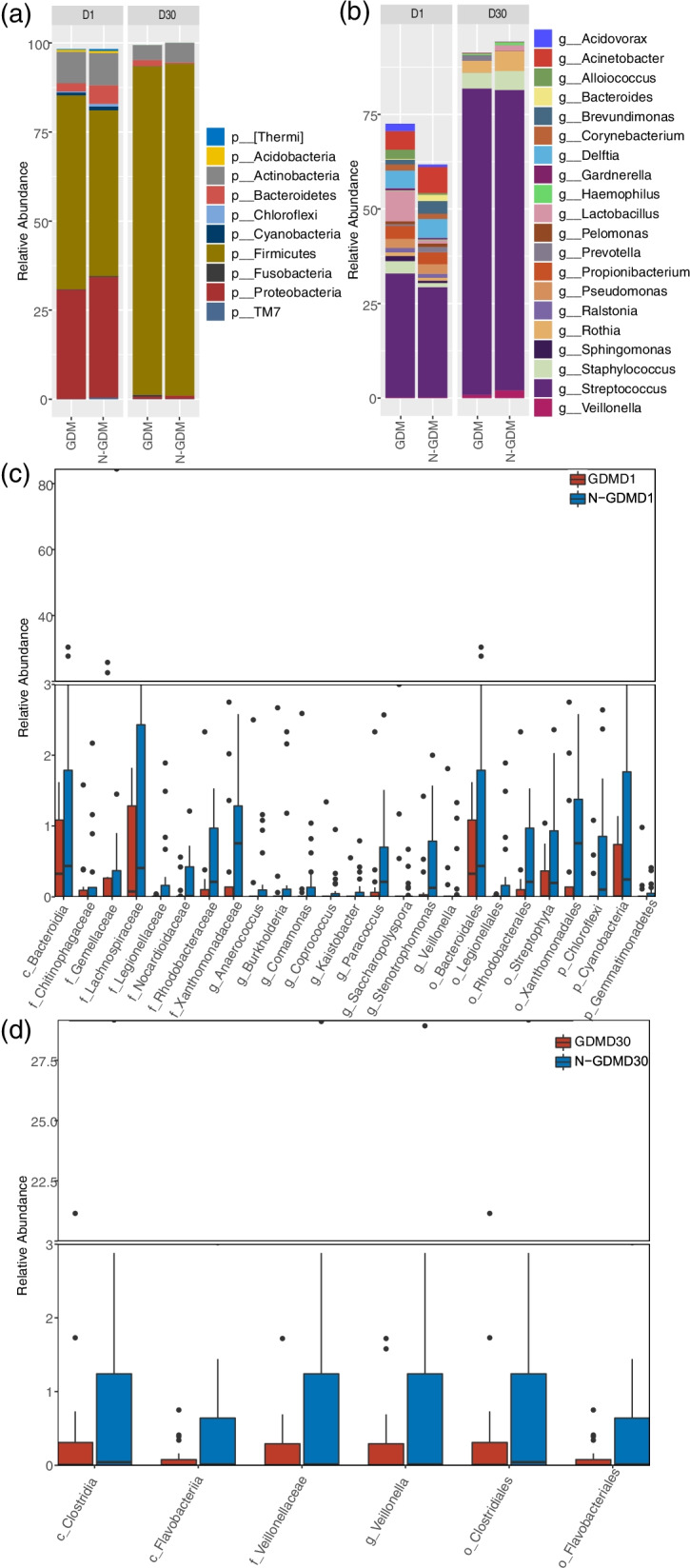
Relative proportions of abundant microbes and their differences between GDM and non-GDM groups. **a** The average relative abundances of abundant phyla in GDM and non-GDM groups in D1 and D30 phases. **b** The average relative abundances of abundant genera in GDM and non-GDM groups in D1 and D30 phases. **c** Relative abundance of differential bacteria between the two groups in the D1 phase. **d** Relative abundance of differential bacteria between the two groups in the D30 phase

The predominant phyla of neonatal oral microbiota included *Actinobacteria, Bacteroidetes, Firmicutes,* and *Proteobacteria* (Fig. 2a), where the sum relative abundance of *Firmicutes* and *Proteobacteria* accounted for more than 80% in both GDM and non-GDM groups in the D1 phase. In the D30 phase, the average relative abundances of *Firmicutes* increased dramatically in both GDM and non-GDM groups, accounting for more than 90%. At the genus level, we showed the average relative abundance > 0.5% (Fig. 2b). *Streptococcus* was the most abundant in both GDM and non-GDM groups in both D1 (32.82% and 29.22%) and D30 phases (81.02% and 79.37%).

In the D1 phase (Fig. 2c), within *Proteobacteria*, we identified that genus *Stenotrophomonas* and the parent family *Xanthomonadaceae* and parent order *Xanthomonadales*, genus *Paracoccus* and the parent family *Rhodobacteraceae* and parent order *Rhodobacterales*, and genus *Comamonas* and *Burkholderia*, were depleted in neonates born to mothers with GDM. Within *Firmicutes*, genus *Coprococcus* and the parent family *Lachnospiraceae*, genus *Anaerococcus* and *Veillonella*, were also depleted in neonates born to mothers with GDM. In the D30 phase (Fig. 2d), genus *Veillonella* and the parent taxa from family to class (*Veillonellaceae, Clostridiales*, and *Clostridia*) within *Firmicutes*, and order *Flavobacteriales* and the parent class *Flavobacteriia* within *Bacteroidetes*, were depleted in infants born to mothers with GDM.

### Functional inference in GDM and non-GDM groups

**Fig. 3:**
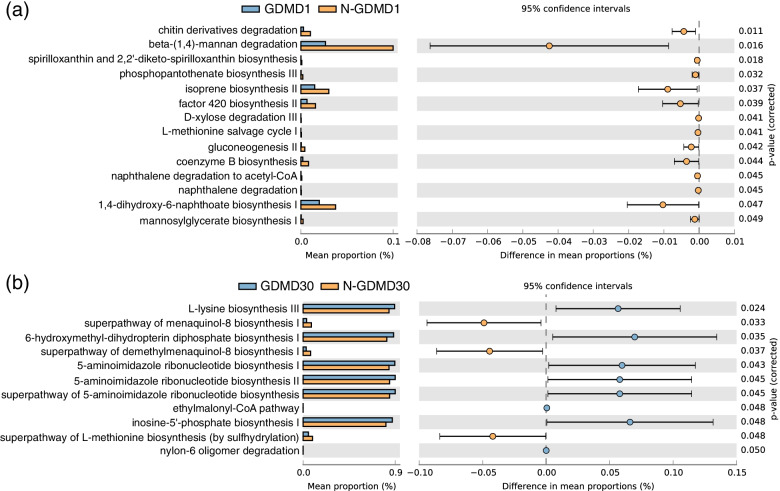
Comparisons of metabolic pathways between GDM and non-GDM groups. **a** The significantly different metabolic pathway between GDM and non-GDM groups in the D1 phase. **b** The significantly different metabolic pathway between GDM and non-GDM groups in the D30 phase

PICRUSt2.0 software was used to do the functional annotation analysis against the MetaCyc database. The differences in metabolic pathways between GDM and non-GDM groups were investigated using STAMP software. The results in the D1 phase showed that pathways PWY-6906 (chitin derivatives degradation), PWY-7456 (beta-(1,4)-mannan degradation), PWY-6581 (spirilloxanthin and 2,2’-diketo-spirilloxanthin biosynthesis), PWY-6654 (phosphopantothenate biosynthesis III), PWY-7391 (isoprene biosynthesis II), PWY-5198 (factor 420 biosynthesis II), PWY-6760 (D-xylose degradation III), PWY-7528 (L-methionine salvage cycle I), PWY-6142 (gluconeogenesis II), P241-PWY (coenzyme B biosynthesis), PWY-6956 (naphthalene degradation to acetyl-CoA), PWY-5427 (naphthalene degradation), PWY-7374 (1,4-dihydroxy-6-naphthoate biosynthesis I), and PWY-5656 (mannosylglycerate biosynthesis I) were significantly enriched in the non-GDM group (Fig. 3a). Moreover, pathways PWY-5838 (superpathway of menaquinol-8 biosynthesis I), PWY-5861 (superpathway of demethylmenaquinol-8 biosynthesis I), PWY-5345 (superpathway of L-methionine biosynthesis) were more abundant in non-GDM group in D30 phase, while PWY-2942 (L-lysine biosynthesis III), PWY-6147 (6-hydroxymethyl-dihydropterin diphosphate biosynthesis I), PWY-6121 (5-amnioimidazole ribonucleotide biosynthesis I), PWY-6122 (5-amnioimidazole ribonucleotide biosynthesis II), PWY-6277 (superpathway of 5-amnioimidazole ribonucleotide biosynthesis), PWY-5741 (ethylmalonyl-CoA pathway), and PWY-6123 (inosine-5’-phosphate biosynthesis I) were significantly enriched in GDM group in D30 phase (Fig. 3b).

### Ecological association network in GDM and non-GDM groups

**Fig. 4:**
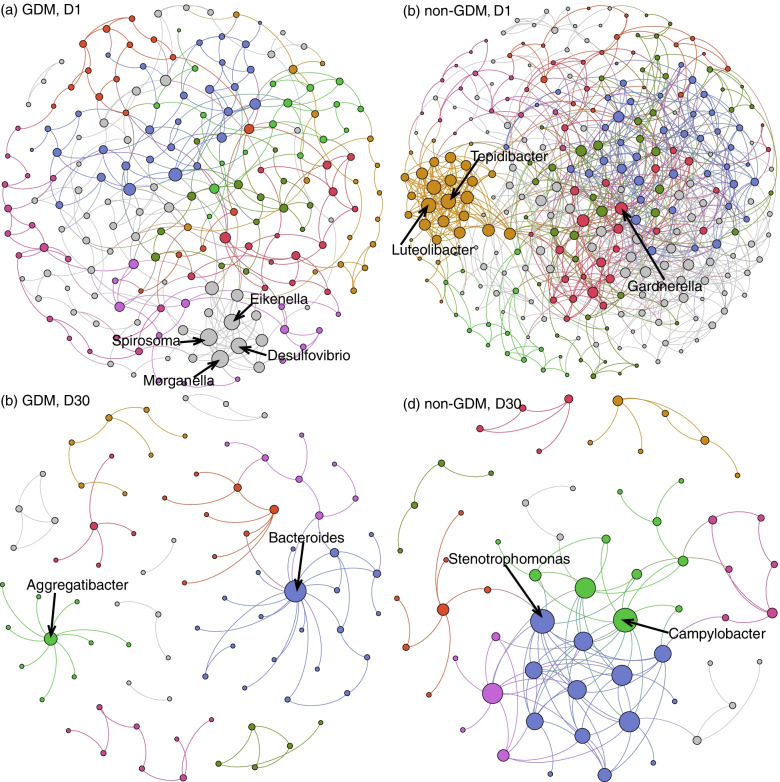
Ecological association network in GDM (**a, c**) and non-GDM (**b, d**) groups in D1 (**a, b**) and D30 (**c, d**) phases, respectively. Each node indicates a genus, and edges between nodes represent their predicted interactions, with larger node indicating more interactions. Different colors indicate different modularity classes

SpiecEasi software was used to construct ecological association networks at the genus level in GDM and non-GDM groups in D1 and D30 phases, respectively. The results demonstrated that the GDM D1 group (Fig. 4a) included 197 nodes and 365 edges. PageRank algorithm indicated the top 5 genera in the GDM D1 group included *Morganella, Spirosoma*, *Desulfovibrio*, *Chryseobacterium,* and *Eikenella*, while 298 nodes and 934 edges were observed in the non-GDM D1 group (Fig. 4b), with the top 5 important genera being *Tepidibacter, Gardnerella*, *Luteolibacter, Anoxybacillus,* and *Bacteroides*. However, in the D30 phase, the number of nodes and edges decreased dramatically in both GDM and non-GDM groups. In the GDM D30 group (Fig. 4c), 85 nodes and 84 edges were observed, and the top 3 nodes were *Bacteroides*, *Aggregatibacter,* and *Achromobacter*. In the non-GDM D30 group (Fig. 4d), 63 nodes and 111 edges existed, and the top 3 nodes were *Campylobacter*, *Stenotrophomonas,* and *Brevibacillus*.

## Discussion

In this study, we examined whether GDM influenced the offspring’s oral microbial colonization, and investigated the change of oral microbiota from birth to 1 month old in a well-controlled, carefully selected cohort of full-term infants without prenatal or postnatal antibiotic exposure. Although no significant differences were observed in alpha and beta diversity metrics between GDM and non-GDM groups, there were distinct differences in oral microbial community structures between D1 and D30 phases. We also found significant differences in the taxonomic composition of oral microbiota from phylum to genus levels between the two groups in both D1 and D30 phases. Metabolic pathways analysis showed that 5-aminoimidazole ribonucleotide biosynthesis and inosine-5'-phosphate biosynthesis were enriched in GDM subjects in the D30 phase. In addition, ecological network analysis revealed apparent differences between GDM and control groups, as well as in D1 and D30 phases, with the non-GDM group and D1 phase containing more high-degree nodes and microbial interactions compared with the GDM group and D30 phase, respectively.

It is well-established that maternal GDM has a long-term influence on the health of their children. However, the influences of GDM on the infant oral microbiota, which is closely related to oral and systemic health in offspring, are less documented. Recently, two studies from different countries have found a distinct oral microbial diversity in neonates born to mothers with GDM [10, 11]. In both studies, a significantly higher alpha-diversity, evaluated with Shannon’s index, in neonates born to mothers with GDM was observed. In the present study, Shannon’s index, Pielou’s index, and observed features value were applied to evaluate the diversity, evenness, and richness of the microbial community. There was no difference in Shannon’s diversity, Pielou’s evenness, or observed features value at any time points. As for beta-diversity visualized through PCoA, we also didn’t find a significant difference between the two groups, while He et al. [11] exhibited a significant separation between GDM and control groups. The inconsistency between our study and previous studies might partly be due to the different sequence processing methods. Mothur pipeline was used to handle and analyze the sequencing data in the study of He and colleagues [11], while in our study, QIIME2 software was applied, which is currently used more commonly. The different methodology may account for the inconsistent findings. More studies are needed in the future to confirm our findings.

To compare the overall oral microbiota composition, we further showed the abundance of taxa in the two groups and performed GLM analyses to identify taxonomic biomarkers that characterize the differences between offspring of mothers with and without GDM. Consistent with previous studies [10, 11], the dominant phyla of neonatal oral microbiota included *Firmicutes, Proteobacteria, Actinobacteria,* and *Bacteroidetes*, accounting for approximately 90%, which was also similar to the composition of gut microbiota [23]. Moreover, significant differences were observed in the taxonomic composition of oral microbiota from phylum to genus level between the two groups, indicating that maternal GDM status would alter oral microbial composition from birth to infancy. Of note, we found that the GDM group exhibited less abundance of *Veillonella* (*P* < 0.001) in the D1 phase, and the difference persisted to the D30 phase (*P* < 0.05). *Veillonella* species within *Firmicutes* is one of the early colonizers of oral microbiota and is prevalent in oral microbiota [24]. Studies have shown that oral *Veillonella* was associated with oral diseases, such as dental caries and periodontal diseases [25–27]. In addition, Crusell et al. [28] reported that during pregnancy, gut *Veillonella* was depleted in women with GDM compared with the non-GDM group. Moreover, the gut *Veillonella* was also depleted in offspring born to mothers with GDM during both the neonatal period and infancy [8]. These studies, together with our findings, indicated that the genus *Veillonella* might be associated with GDM, and there might be some transmission paths from the maternal gut microbiota to neonatal oral and gut microflora. However, hitherto limited information is known about the mechanisms underlying the link between *Veillonella* and GDM.

Using metagenome function prediction, we found that 5-amnioimidazole ribonucleotide biosynthesis pathways were significantly enriched in GDM group in D30 phase. 5-aminoimidazole ribonucleotide (AIR) is a key intermediate of purine nucleotide and thiamine biosynthesis [29]. In addition, inosine-5'-phosphate biosynthesis pathway also showed the enrichment significance in saliva of infants born to GDM mothers. It's worth noting that the 5-aminoimidazole ribonucleotide biosynthesis pathway and inosine-5'-phosphate biosynthesis pathway make up the purine nucleotides de novo biosynthesis pathway, which plays an essential role in many cellular processes, including DNA replication, transcription, cellular signaling, and energy metabolism [30]. A recent study of neonatal gut microbiota also found that pathways related to carbohydrate and nucleotide metabolism were enriched in neonates born to GDM mothers [6]. Our study, together with previous studies [6, 11], indicated that maternal GDM might promote the succession of high-energy-providing microbiota with altered metabolism in their offspring. Consequently, maternal GDM could further mediate the development of macrosomia and childhood metabolic diseases such as obesity in later life.

GDM and non-GDM groups harbor distinct oral microbial ecological association networks, as well as the D1 and D30 phases. Ecological association networks showed more microbial correlations in the non-GDM group than the GDM group. We found that *Gardnerella* was an important genera in the non-GDM D1 group. It is known that *Gardnerella* was considered as one of the causes of bacterial vaginosis [31]. Previous studies also suggested that *Gardnerella* may change the host landscape in a way that makes other organisms more likely to colonize or cause disease [31]. However, the study of Lukic et al. [32] did not support a more prevalent vaginal infection by *Gardnerella* in diabetic women. In addition, some top genera observed in the GDM group, such as *Morganella* and *Eikenella*, were not found in the non-GDM group. The genus *Desulfovibrio*, which was previously found to be enriched in patients with GDM or type 2 diabetes [28, 33], also had more microbial interactions with other colonizers in the GDM group in our study. The difference in ecological networks between the GDM and non-GDM groups reflected maternal GDM might disrupt the ecology of oral microbiota in offspring. Meanwhile, the high-degree nodes and microbial interactions decreased dramatically in the D30 phase compared with the D1 phase, regardless of the GDM group or non-GDM group, indicating the oral microbiota has changed greatly over time. At birth, the neonatal oral microbiota was mainly derived from the maternal intrauterine environment, and influenced by vaginal or skin microbiota, while at 1 month of age, with the introduction of breast milk or formula, the infant oral microbiota changed dramatically [13, 34].

### Strengths and limitations

One highlight of our study is the well-controlled, hypothesis-directed cohort study design. All subjects were full-term infants without prenatal or postnatal antibiotic exposure, and there were no significant differences in general characteristics between the GDM and non-GDM groups, eliminating the major confounders that typically interfered with early infant oral microbial colonization. Moreover, we not only investigated the influences of GDM on neonatal oral microbiota, but also followed up to 1 month old; therefore, for the first time, we explored the longitudinal changes in the oral microbiota composition from birth to infancy according to GDM status.

However, there were also certain limitations needed to be considered. Firstly, the sample size was limited. However, as mentioned above, the general characteristics were similar in the two groups, minimizing the influence of confounding factors on the results. Secondly, a causal relationship between maternal GDM and oral microbiota in offspring cannot be confirmed by the present study design. In addition, all participants in the current study were Han Chinese from one hospital. Given that the oral microbiota varies among different races, results of the present study cannot be directly transferred to other ethnicities. Further studies should be performed in other populations to validate and extend our findings.

## Conclusions

In conclusion, our study demonstrated that maternal GDM was associated with different colonization of oral microbiota in neonates, although the distinct difference between GDM and non-GDM groups diminished in infancy. The oral microbiota structure, composition, functions, and ecological networks changed dramatically over time, regardless of GDM or non-GDM group. Further research in large, prospective, birth cohorts with diverse biological samples from both mothers and children will be necessary to understand how maternal GDM affects the early oral microbiota changes, and consequently, oral and systemic health in their children.

## Data Availability

Sequencing reads and metadata were deposited as entire raw data in the National Center for Biotechnology Information Sequence Read Archive (NCBI SRA BioProject ID: PRJNA760654). https://www.ncbi.nlm.nih.gov/bioproject/PRJNA760654/
